# Beware of counter-intuitive levels of false discoveries in datasets with strong intra-correlations

**DOI:** 10.1186/s13059-025-03734-z

**Published:** 2025-08-18

**Authors:** Chakravarthi Kanduri, Maria Mamica, Emilie Willoch Olstad, Manuela Zucknick, Jingyi Jessica Li, Geir Kjetil Sandve

**Affiliations:** 1 Scientific Computing and Machine Learning Section, Department of Informatics, University of Oslo, Oslo, Norway; 2 UiORealArt Convergence Environment, University of Oslo, Oslo, Norway; 3 PharmacoEpidemiology and Drug Safety Research Group, Department of Pharmacy, Faculty of Mathematics and Natural Sciences, University of Oslo, Oslo, Norway; 4 Department of Biostatistics, Faculty of Medicine, University of Oslo, Oslo, Norway; 5 Department of Statistics and Data Science, University of California, Los Angeles, CA USA

**Keywords:** False discovery rate (FDR), Benjamini-Hochberg (BH), Multiple testing adjustment, High-dimensional omics data

## Abstract

The false discovery rate (FDR) controlling method by Benjamini and Hochberg (BH) is a popular choice in the omics fields. Here, we demonstrate that in datasets with a large degree of dependencies between features, FDR correction methods like BH can sometimes counter-intuitively report very high numbers of false positives, potentially misleading researchers. We call the attention of researchers to use suited multiple testing strategies and approaches like synthetic null data (negative control) to identify and minimize caveats related to false discoveries, as in the cases where false findings do occur, they may be numerous.

**Supplementary Information:**

The online version contains supplementary material available at 10.1186/s13059-025-03734-z.

## Background

With high-throughput data generation and data-driven analysis becoming increasingly prevalent across biology, the simultaneous statistical testing of multiple hypotheses has become a standard technique in many biologists’ toolboxes. The selection of hypotheses (e.g., for detailed investigation) is often done by thresholding based on *p*-values. Even when all the null hypotheses are true, a controlled proportion of the hypotheses (known as alpha, traditionally set to 5%) will be falsely rejected [1]. Various adjustment methods have thus been proposed to control false discoveries in settings where multiple tests are systematically explored. These methods are known to adequately control either the family-wise error rate (FWER—the chance of reporting any false finding) or the false discovery rate (FDR) the expected proportion of false discoveries among all reported findings. Formally, FDR is the expectation of the False Discovery Proportion (FDP), defined as the ratio of the number of false discoveries to the total number of discoveries (ensuring at least one discovery to avoid division by zero). Since both values vary across datasets, FDP is a random variable. In frequentist statistics, FDR is the expectation of FDP over potential datasets drawn from the same distribution as the dataset at hand, depending on the needs of the study [2]. Although findings from data-driven exploration are usually followed up with independent verification, this comes with a cost, comes with a delay, and is not always possible. It is thus highly desirable to limit false findings, which is what properly controlled (multiple) statistical testing aims to achieve. The choice between FWER and FDR has long been a trade-off between type I and type II error rates. Analysts often understand this as a choice between “highly conservative” and “less conservative” correction methods [2–4]. When faced with the dilemmas of other study design considerations (e.g., power, biases) that may limit the detection of “statistically significant” findings, many analysts choose “less conservative” methods under the premise that the FDR methods still control the expected FDP at a pre-specified level, leading to a controlled risk and cost of false findings [2, 5, 6].

However, it is well known that the proportion of falsely rejected null hypotheses may increase beyond its formally controlled level depending on other factors like broken assumptions for the statistical test, study biases, or the researcher’s flexibility in analyzing the data [7]. When analyzing the statistically significant findings (post-correction), analysts may thus be aware that the proportion of false findings could easily be somewhat higher than the reported FDR but presume that as long as there was no p-hacking [8] and the assumptions are only slightly broken (e.g., only slight presence of study biases), the large majority of their observed findings will still reflect genuine effects. Specifically, suppose a large number of findings are reported after following a multiple testing procedure. In that case, analysts may follow the intuition that if, for example, hundreds of genomic sites are reported as findings, they cannot all be false findings. In certain biological contexts—like differential gene expression, pathway enrichment, and epigenome-wide association analyses—this could be taken to imply the presence of an underlying biological mechanism that involves at least some of the many findings (e.g., genes or genomic sites) being reported. This could result in either futile validation experiments or the scientific literature being plagued with false findings.

Contrary to this intuition, we show in this manuscript that when strong dependencies exist between the many hypotheses being tested, such a central intuition regarding FDR control does not hold (see Additional file 1: Fig. S1 for a graphical summary). In fact, even though a positive correlation between tests is (rightly) considered safe for FDR controlling procedures like Benjamini-Hochberg (BH) [9], in the sense that it does not break the formal guarantees of the procedures, it can still lead to counter-intuitive results: in combination with slight data biases, broken test assumptions, publication bias (or even just rare coincidence alone), or the researcher’s flexibility in analyzing the data, hypothesis dependencies may (as demonstrated later in the article) lead to thousands of sites along the genome being falsely reported, even when all the null hypotheses are true.

## Results and discussion

**Fig. 1:**
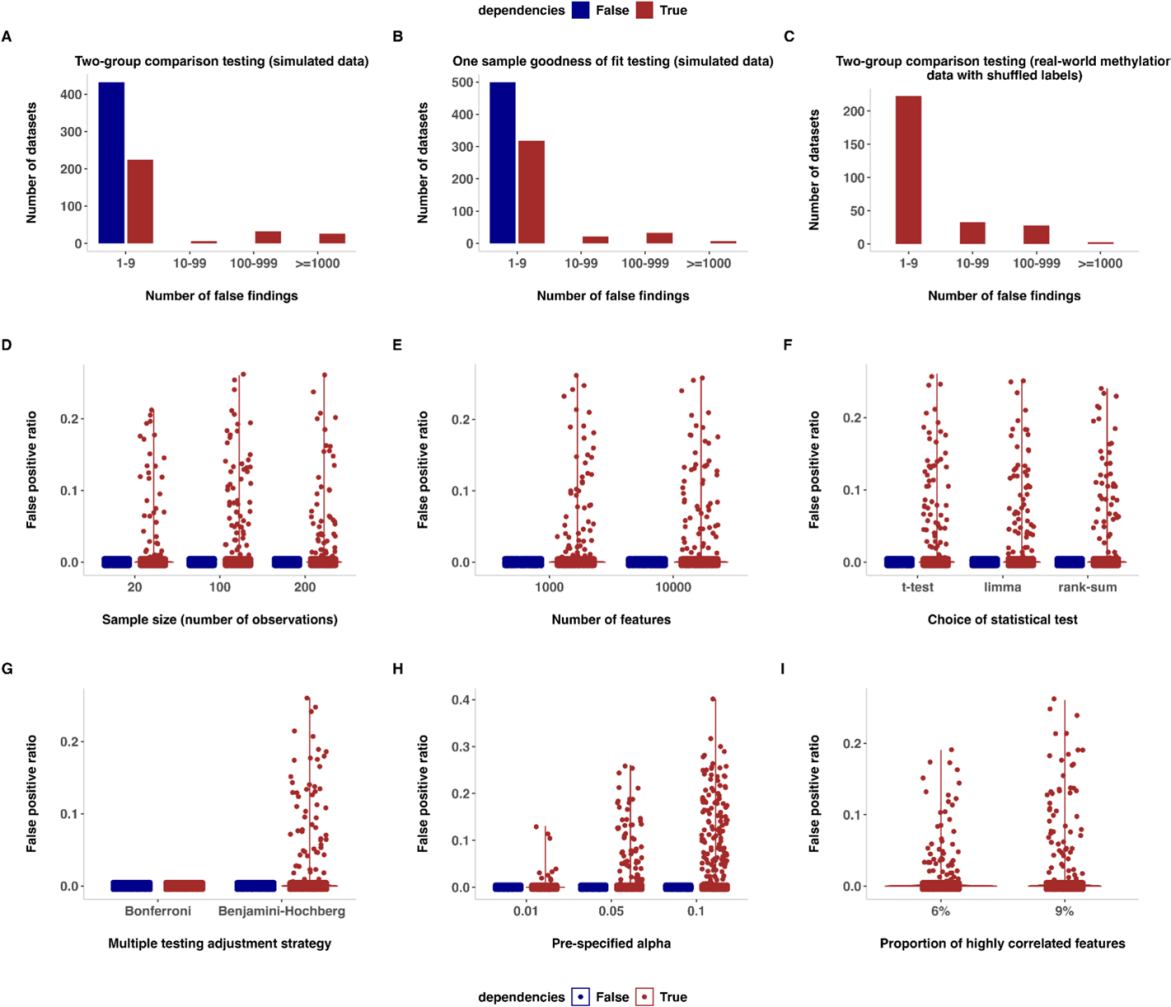
**A**–**C** Conditional histograms (conditioned on number of false findings being greater than zero) showing the distribution of the number of false findings across datasets (10,000 datasets assessed in each sub-panel) after performing feature-wise statistical hypothesis testing with the standard two-group *t*-test that assumes equal variance (**A**, **C**) or the KS test assessing whether a set of features is sampled from a shared underlying normal distribution (**B**) followed by BH FDR correction (FDR nominal control at 0.05). Note that bins representing zero false findings have been excluded for improved readability in panels **A**–**C** A total of 10,000 datasets each for Beta (**A**) and Normal (**B**) distributions have been simulated with 100 observations across 10,000 features with and without dependencies (shown in different colors). In the simulated datasets with dependencies, the proportion of highly correlated features was similar to real-world experimental omics data in life sciences. Additionally, we analyzed the phenomenon for real-world methylation array datasets (**C**) with shuffled labels. **D**–**I** Boxplots illustrating the impact of specific study design characteristics or analytical choices on false findings across all 10,000 analyzed datasets with and without dependencies. Note that the false discovery proportion (FDP) is always 100% in all figure panels because all null hypotheses are true. We, therefore, plot the false positive ratio (FPR), which is defined as the proportion of true negatives that were falsely categorized as positive. We demonstrate the impact of the sample size (**D**), number of features (**E**), type of statistical test (**F**), FDR/FWER correction method (**G**), level of significance (alpha) (**H**), and the total proportion of correlated features (**I**). Note that here, the statistical hypotheses testing performed was a two-group *t*-test followed by BH correction at 5% FDR level. The number of observations was fixed at 100, and the number of features remained constant at 10,000 except when they were varied for investigation. For similar findings on one-sample tests assessing whether a set of features is sampled from a shared underlying normal distribution, see Additional file 1: Fig. S2. Note that the FDR, which is the average of FDP across all the 10,000 datasets in each sub-panel (**A**–**I**), was still controlled under 5% in both the settings with and without dependencies (i.e., there was no inflated FDR); however, in the presence of dependencies, a high FPR was observed sporadically

In this study, we first analysed FDR control in two settings with all null hypotheses being true: (a) when high-dimensional datasets contain correlated features and (b) when they do not. For this, we used both simulated and real-world datasets of DNA methylation arrays (~ 610,000 unique datasets; see Methods for details). To show that the findings are not connected to the intricate details of any one specific type of statistical test, we conducted experiments for two different statistical testing settings: (a) comparing the means of two different groups and (b) assessing whether a set of features are sampled from a shared underlying normal distribution. Irrespective of the type of statistical hypothesis testing, and on both simulated and real-world datasets, we observed an increased frequency of observing a very high number of false findings (as high as 20% of the total number of features) when the datasets contained correlated features (Fig. 1 A–C). Note that the FDR (and equivalent FWER because all null hypotheses are true) was still controlled according to its formal guarantee—i.e., the procedure resulted in zero reported findings in > 95% of cases. However, in the remaining < 5% of cases, the number of reported sites was, at times, very high. This phenomenon of sometimes observing a high number of false findings persisted when using BH correction [9] at the routinely used predefined FDR nominal levels (e.g., 5%, 10%), irrespective of the sample size (number of observations), the total number of features, and the choice of statistical tests (different types of parametric or non-parametric tests) (Fig. 1D–H, Additional file 1: Fig. S2 A–E), as long as the datasets mimicked the real-world datasets in terms of the degree of correlations and the proportion of correlated features (Additional file 1: Table S1). Note particularly that the FDP is always 100% in the settings we analyse because all null hypotheses are true from the outset. The variance of the number of rejected features per dataset was larger for correlated tests than under independence (where it would follow a binomial distribution). This was the case for any alpha level (trivially including Bonferroni correction [10–12]). The BH correction [9] leads to a further exaggerated increase in variance (Additional file 1: Fig. S3). Although the effect started to gradually decrease when the total proportion of correlated features was lower than what is observed in some of the real-world datasets from life sciences, we observed that the phenomenon still persisted (Fig. 1I, Additional file 1: Fig. S2F).

**Fig. 2:**
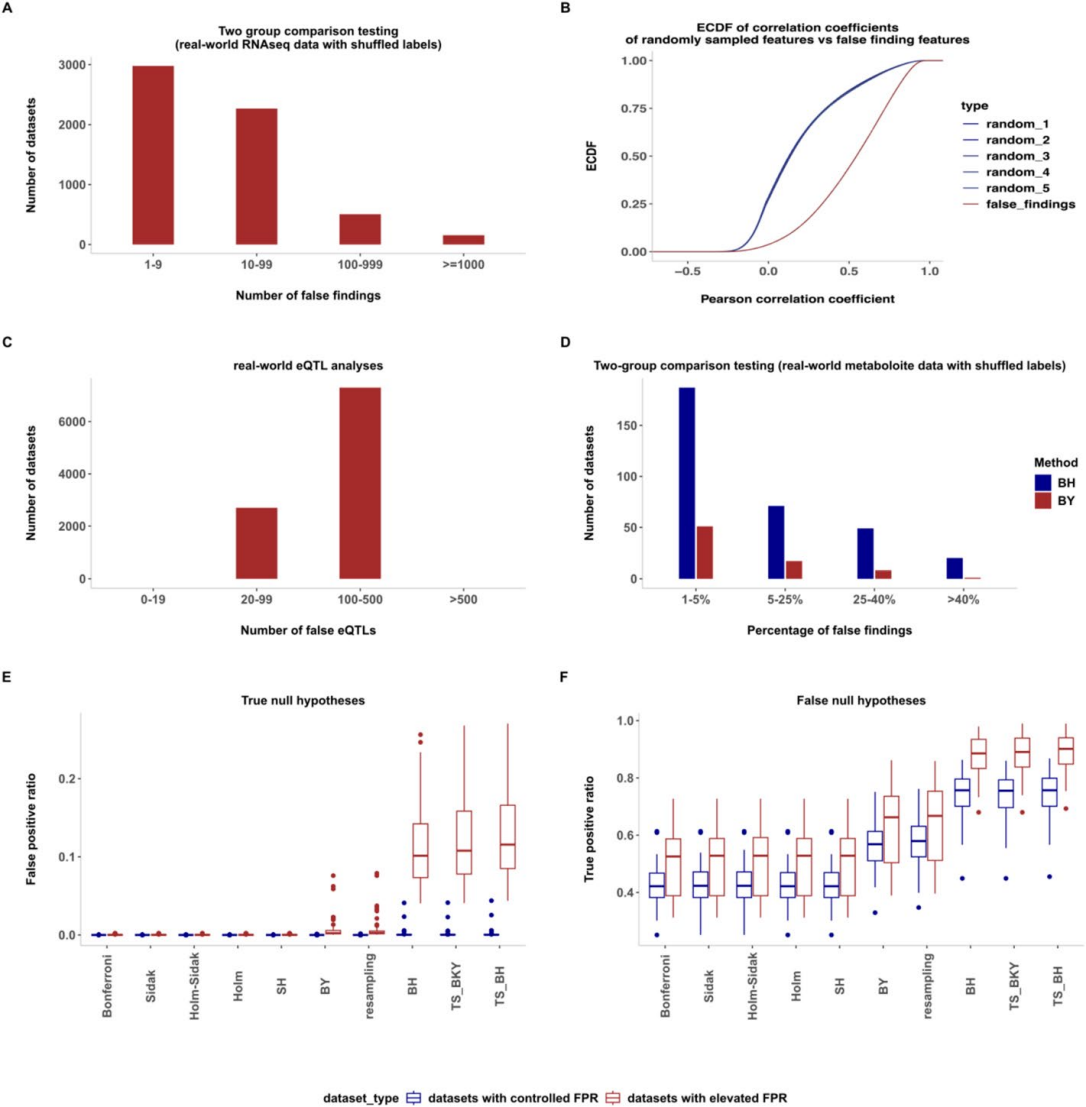
**A** Conditional histograms (conditioned on the number of false findings being greater than zero) showing the number of datasets (*y*-axis) with a certain number of false findings (on *x*-axis) across real-world RNA-seq datasets with shuffled labels (10,000 datasets). Feature-wise statistical hypothesis testing with standard two-group comparison was performed using DESeq2, followed by BH FDR correction (FDR nominal control at 0.1). Note that bins representing zero false findings have been excluded for improved readability. The datasets contained ~ 40,000 features. **B** Empirical cumulative distribution plots of the correlation coefficients. The distribution of the upper triangle of the Pearson correlation coefficient matrix for all false findings in a specific dataset with many false findings is depicted in a distinct color. In contrast, dark blue-ish colours represent the distribution of the upper triangle of the Pearson correlation coefficient matrix for randomly sampled features of the same size, repeated five times, from the same dataset. **C** Histograms showing the number of datasets (*y*-axis) with a certain number of false findings (on *x*-axis) across real-world eQTL datasets with shuffled gene expression levels (10,000 datasets). eQTL analysis was carried out using the MatrixEQTL R package [24] using linear models, followed by default FDR correction using the BH method at a FDR nominal control of 0.05. **D** Conditional histograms (conditioned on the number of false findings being greater than zero) showing the number of datasets (*y*-axis) with a certain percentage of false findings (on *x*-axis) across real-world metabolite datasets with shuffled labels (10,000 datasets). Feature-wise statistical hypothesis testing was performed using a two-sided *t*-test with the null hypothesis that there is no difference between the group means of two randomly assigned groups, followed by BH correction with a FDR nominal level of 5%. The colour indicates the findings using either the BH or BY correction methods. **E** FPR comparison for different multiple testing adjustment approaches, where all the null hypotheses were true. **F** True positive ratio (TPR) comparison for different multiple testing adjustment approaches for null hypotheses that are false. A subset of datasets with controlled FPR and elevated FPR were selected from previous analyses shown in Fig. 1G. for analyses shown in **E**–**F**

To demonstrate that the implications of our findings extend to any type of data with correlated features, we repeated the experiment of analysing FDR control where all null hypotheses are true in real-world gene expression data, metabolite data, and eQTL data analyses. For gene expression data, we used real-world bulk RNA-seq datasets with shuffled labels (~ 10,000 unique datasets with ~ 40,000 features; see the “ Methods” section for details). We performed the standard differential expression analysis comparing groups for expression differences using DESeq2 [13], followed by BH correction (which also happens to be the default option in DESeq2). We used an FDR nominal level of 10%, as such a level is commonly used in gene expression studies. Similar to the observations on simulated and real-world methylation data (Fig. 1A–C), we again observed an increased frequency of a high number of false findings in real-world RNA-seq data (Fig. 2A). We randomly picked one of the datasets with an elevated false positive ratio (FPR) as a representative example and observed that the false-finding features were highly correlated with each other when compared to randomly drawn features from the same dataset (Fig. 2B).

For metabolite data, we used real-world metabolite datasets with shuffled labels (10,000 datasets with ~ 65 features; see the “ Methods” section for details). We performed a two-sided *t*-test with the null hypothesis that there is no difference between the group means of two randomly assigned groups, followed by BH correction with a FDR nominal level of 5%. We again observed an increased frequency of a high number of false findings (Fig. 2D). Although the FDR is still controlled (with zero false findings in ~ 96% datasets), when false findings occurred, the phenomenon described above is even more pronounced in real-world metabolite data, where sometimes as high as ~ 85% of the total features were found to have significant differences between groups. This observation can be attributed to the higher degree of dependencies known to be present in metabolomics data.

Multiple hypothesis testing is also a major issue in fields involving genetic variation. Genome-wide association studies (GWAS) have long used Bonferroni correction with a threshold of 5 × 10^−8^ (corresponding to a 0.05 threshold per a million sites tested) [14], but permutation testing is also increasingly being considered the gold standard [15] because of the well-known correlations along the genome, especially due to linkage disequilibrium (LD). Quantitative trait locus (QTL) studies associating genetic variation with traits like gene expression (eQTL) [16], DNA methylation (mQTL) [17], and metabolites (metQTL) [18] also face a similar issue of multiple testing with dependent tests. Conscious of the dependencies arising from pervasive LD along the genomes, the QTL field has increasingly focused on developing and implementing LD-aware multiple testing corrections. This includes emphasis on efficient locus-restricted permutation testing and hierarchical procedures that often involve local permutation testing [19–22]. Huang et al. [23] performed a comprehensive evaluation of various multiple testing strategies across study design considerations of eQTL studies and showed that global FDR correction methods like BH are “inappropriate for eQTL studies, as they give inflated (sometimes substantially) FDR that worsen as sample size increases”. We observe similar findings in our eQTL experiments, where we used real-world eQTL data with shuffled gene expression levels (10,000 datasets; see Methods for details). We performed standard eQTL analyses using linear models from the famous MatrixEQTL package [24] with population stratification as covariate (cited ~ 1800 times as of June 2025, with the BH method as the default for FDR). We observed a substantially increased incidence of a high number of false findings (Fig. 2C), much more frequently than any other type of data analyzed in this study. Huang et al. [23] observed that the most commonly used hierarchical approach in eQTL studies (e.g., also used by GTEx [25]; permutation testing for local correction, followed by a global correction using BH or similar) is better at controlling FDR. However, based on our findings from all the above experiments, we urge the readers to be aware that FDR correction methods like BH [9] can, in some cases, report very high numbers of false positives.

### Comparison of false and true positive ratios of multiple FDR and FWER control methods

FDR control in the presence of correlated features is a known problem, and various approaches have been proposed that operate under a range of assumptions regarding the dependence structure. We compared the behaviour (FPR and TPR) of some of the well-known approaches that have been suggested to be robust in controlling FWER or FDR in the presence of positively correlated test statistics. To accomplish this, we expanded the comparison from Fig. 1G by incorporating additional methods. Instead of using all the 10,000 datasets that were previously used in Fig. 1G, we used only ~ 160 datasets. Particularly, we used all those simulated datasets where the FPR was > 0.05 (~ 60 such datasets, hereafter referred to as datasets with elevated FPR) and a random subset of 100 datasets where the FPR was < 0.05 at a pre-defined FDR nominal level of 5% using the BH method in previous analyses (hereafter referred to as datasets with controlled FPR). The reason for this restriction is the computational expense of resampling-based FDR controlling procedures, where we intended to have at least 1000 permutations for each dataset.

Beyond the BH [9] and Bonferroni [11] methods previously examined (Fig. 1G), we included the following FWER or FDR controlling approaches: Sidak [26], Holm [27], Holm-Sidak [26, 27], Simes-Hochberg (SH) [28, 29], Benjamini-Yekutieli (BY) [30], two-stage approaches based on the work of Benjamini-Hochberg (TS_BH) [31], Benjamini, Krieger and Yekutieli (TS_BY) [32], and a resampling-based FDR controlling procedure similar to that of Yekutieli and Benjamini (resampling) [33]. To throw light on the TPR of all the FDR control procedures simultaneously with the FPR, in each dataset, we simulated 1–2% of the total hypotheses being tested as false. For this, we used exactly the same datasets as before but now introduced effect sizes for a subset of features that align with a realistic number of significant hits reported in typical experimental studies.

Regarding the FPR (Fig. 2C), FWER methods (Bonferroni, Sidak, Holm, Holm-Sidak) and one FDR method (SH) had consistently low FPRs. FDR methods (BY, resampling-based, BH, TS_BKY, TS_BH) showed varied behavior. The BH [9], TS_BKY [32] and TS_BH methods had many instances of high FPRs. The BY method [30] and the resampling-based approach [33] showed fewer instances of a moderate level of FPRs, albeit less frequently and less severe than other FDR methods (Fig. 2C).

Concerning the TPRs (Fig. 2D), FDR methods BH [9], TS_BKY [32] and TS_BH [31] resulted in the highest TPRs. In contrast, the FWER methods and one FDR method (SH) showed the lowest TPRs. BY [30] and the resampling-based approach [33] showed a moderate level of TPRs. Collectively considering FPRs and TPRs, the methods can be categorized into three classes: (a) those that resulted in the lowest FPRs at the expense of the lowest TPRs, (b) those that had the highest TPRs but many instances of high FPRs, and (c) those that had moderate TPRs with fewer, less severe instances of FPRs. These observations align with the findings of a previous benchmarking effort [34], where the BY method [30] has been shown to be conservative in controlling the FDR way below the specified 5% nominal level, often controlling less than 1%, at the expense of loss of TPR that was shown to be 30% lower than that of the BH procedure [9].

### BH method remains a popular choice even in the presence of correlated features

While several methods have been proposed to address FDR control in the presence of correlated features, the persistent challenge lies not in the absence of solutions but in practical adoption, potentially owing to the type II error rates of suited procedures. Popular tools designed for performing multiple tests for gene expression data (e.g., DESeq2 [13], edgeR [35], limma [36] that are together cited > 150,000 times) have the BH procedure as the default option for controlling FDR. These tools do not provide a rationale for the choice of BH procedure, except the following statement in the user guide of limma: “Benjamini and Hochberg’s control of the false discovery rate assumes independence between genes, although Reiner et al. have argued that it works for many forms of dependence as well.” Notably, we demonstrated using real-world gene expression data that there can sometimes be numerous false findings with BH, and we showed a high degree of correlation between false findings (Fig. 2A–B). To further investigate how prevalent the BH procedure is in the presence of dependent test statistics, we performed a literature search in eight different journals that are popular avenues for omics-based investigations. We particularly focused on DNA methylation studies published from 2020 through March 2025 and analysed the prevalence of three multiple testing adjustment procedures: BH [9], Bonferroni [11], and Benjamini-Yekutieli [30]. The search process is detailed in the “ Methods” section. ~ 60% of the relevant articles (that performed multiple statistical tests and mentioned multiple testing adjustments) did not explicitly mention any of the selected adjustment methods. Of the remaining relevant articles across journals that mentioned any of the selected adjustment methods, ~ 57% of all the articles used the Bonferroni procedure, followed by ~ 31% usage of the BH procedure, compared to < 2% that used the Benjamini-Yekutieli procedure (Additional file 1: Table S2). These findings reasonably reflect the prevalence of the BH procedure in studies with dependent test statistics. Overall, these statistics and BH being a default option in popular libraries suggest that the BH procedure remains a popular choice even in the presence of correlated statistics.

### Recommendations and outlook

When an analysis results in a large number of significant hits, it may be intuitive to conclude that at least some of them must be true—i.e., that the result is a robust indication of some form of underlying biology. To identify whether an observed high number of significant hits could occur merely by chance (whether such a number is consistent with all nulls being true, due to strong feature dependence), the following two approaches could be of relevance: (a) the distribution of correlation coefficients between significant hit features can reveal if the findings can be potentially attributed to correlations between features, (b) a distribution of the number of significant findings based on repetitions of negative control data (similar to “negative controls” used by experimental biologists [37]) can reveal if a similar counter-intuitively high number of false findings can occur given the properties of the original dataset. Here, simple permutations of the labels of the observations in the original dataset could be a good starting point for generating in silico negative control data and can suffice for the analysis of differences between groups. However, the permutation of labels may not suffice to generate a valid null distribution in certain settings (e.g., when the effect of interest involves changes in trajectories and spatial organization). In such cases, sophisticated models that are either generic or domain-adapted (like being specific to particular omics types or a particular technology (e.g., single cell)) could be used to generate synthetic null data. For instance, knockoffs [38–40] are a class of methods that can generate synthetic null data while preserving the intrinsic dependence structure of the original datasets. The knockoffs require knowledge of the joint distribution of features and are computationally intensive because of the strict requirement of exchangeability, where swapping any subset of features with their knockoff counterparts requires preserving the joint distribution of all the original features and knockoffs. Novel frameworks such as SyNPar [41, 42] have been proposed recently to generate synthetic null data efficiently and easily by overcoming the challenges of knockoffs. Generic synthetic data generation libraries (e.g., [43] also include copula-based models and deep generative models that can preserve either the dependence structure and/or the marginal statistical properties of the datasets. Domain-adapted synthetic data generation libraries (e.g., scDesign3 [44] for single-cell data) have also been developed to preserve both the dependence structure and marginal statistical properties of the datasets. Song et al. [45] demonstrate one example of using domain-adapted synthetic data generation libraries to generate synthetic null data to minimize FDR. Irrespective of the choice of the tools/methods used, valid synthetic null data should be able to nullify the effect of interest under the null hypothesis. As an example of synthetic null data generation that involves more than the permutation of labels, we provided a short tutorial using a generic synthetic data generation library in the accompanying GitHub repository.

Making a single recommendation that fits all goals is challenging when addressing the identified phenomenon, as the tolerance for various types of error rates often influences the choice of a suited FDR controlling procedure in the presence of dependencies between test statistics. Nevertheless, the following would be our recommendation based on the findings: Although no instances of a high FPR were observed with Bonferroni and similar FWER approaches, we would not suggest making them a general recommendation, owing to low TPRs (unless high type II errors can be tolerated). Among the FDR methods that had the highest TPRs, the popular BH method had, on average, the lowest FPRs (Fig. 2C–D). Notably, the BH method still controls the FDR according to its formal guarantee, which was evident in all the analyses of Fig. 1, where zero false findings were observed in > 95% of the cases. One could thus still use the BH method in settings with high feature dependence. However, to ensure correct interpretation, users need to understand the formal guarantees of the BH method precisely. Specifically, with the BH method we would advise both analysts and peer reviewers to take into account that even a very large number of significant hits does not provide a sure indication of underlying biology—with strong dependency, a large number of false findings could occur just by chance (even if all null hypotheses hold). Finally, although the BY method does not completely eliminate the described phenomenon, it makes it much less frequent and less severe. As long as a slightly increased level of type II error can be tolerated, we therefore recommend the BY method as a good compromise that reduces the risk of unwarranted interpretations while still providing good statistical power.

The widespread adoption of the BH method in omics data analysis may stem from several factors. Computationally, it offers a less conservative and powerful alternative to methods like BY while avoiding the high computational cost of permutation testing. Its prevalence as a default option in many popular omics data analysis libraries also contributes to its frequent, and sometimes uncritical, application. Analysts may also strategically select BH to balance Type I and Type II error rates, or they may be aware that its FDR control properties hold when test statistics are stochastically independent or satisfy positive regression dependency—a condition often met in omics data, as supported by our findings of zero false findings in over 95% of cases. Another plausible factor contributing to the less-informed application of the BH method is the lack of explicit documentation in widely used statistical software, such as R’s “p.adjust()” function and other omics-specific libraries, regarding the underlying dependence structure assumptions required for BH to control FDR. Particularly, FDR control by BH holds under the so-called positive regression dependency condition introduced by Benjamini and Yekutieli (2001) [30]. It says that for any increasing set *D* of *p*-value outcomes, conditioning on one null *p*-value being smaller (i.e., more significant) cannot decrease the probability that the overall outcome falls in *D*. Intuitively, it implies that conditioning on one null *p*-value becoming more significant does not decrease the probability of other null p-values becoming less significant. While our results indicate that the BH method often maintains FDR control in omics data, even with high feature dependence, it is crucial for analysts to be aware that a large number of significant findings does not automatically guarantee underlying biological relevance. This highlights the need for clearer methodological guidance and transparent reporting of FDR control choices in scientific literature.

## Conclusions

In summary, our findings demonstrate that with highly correlated datasets, as in omics and life sciences, FDR correction methods like BH [9] can, in some cases, report very high numbers of false positives. This can lead to thousands of falsely reported findings, even if all null hypotheses are true. It can be highly counter-intuitive that these findings may all be false, which can mislead researchers to conclude on the presence of an underlying mechanism (or signal) that involves at least some of the reported sites. An underlying mechanism’s alleged existence may indeed be a given study’s main conclusion. Slight data biases, broken test assumptions, researcher degree of freedom, or publication bias may further compound this effect. Thus, we call the attention of researchers using multiple statistical tests in high-dimensional data to be aware of dependencies in the datasets, to use suited multiple testing strategies, and to beware that in cases where false findings do occur, they may be numerous.

## Methods

### Synthetic and real-world datasets

#### High-dimensional data with a large degree of dependencies

DNA and its properties are examples of high-dimensional data having substantial dependencies between features. In this study, we used DNA methylation data as one example of high-dimensional datasets with correlated features to investigate its impact on FDR control. For this, we use both simulated and real-world methylation array data. Methylation arrays have remained a popular technology for assaying genome-wide DNA methylation patterns. Methylation array data is typically known to follow the beta distribution, and the beta values are generally assumed to be biologically interpretable. The beta-distributed methylation values are often transformed to *M* values (logit transformation) before any statistical testing, owing to their more convenient statistical properties. A frequent need for univariate statistical testing on methylation data arises from the hypothesis that a fraction of the methylation sites differ between two groups of observations (e.g., disease vs healthy), where the null hypothesis is often that the methylation levels do not differ between groups (a two-group statistical test). A fraction of the null hypothesis could be false when there are true biological effects. In this study, we chose to investigate the FDR control when all null hypotheses are true using both synthetic and real-world methylation data. Thus, neither the simulated nor the real-world datasets that we used contained any known signal that differentiated the two groups being compared.

#### Simulated methylation array data

In this study, we chose to investigate the FDR control when all null hypotheses are true. Thus, we do not introduce any signal into the methylation data in a large majority of the settings (see point 3 below for an exception). For simulating data that mimics real-world methylation data, we used approaches that are often referred to as “plasmode simulation” [46–50], where some aspects of the data-generating model are known and used for simulations (e.g., using estimated distributional properties of features in observed data, using estimated associations between features in observed data).

1. To simulate methylation array data mimicking the correlation structure from real-world experimental datasets, we followed the procedure described below:
  - We randomly sampled the desired number of methylation sites from a real-world experimental dataset (specifically, Illumina Infinium EPIC Human methylation array data from GEO accession number: GSE161651 [51, 52]) and estimated their beta distribution parameters (alpha and beta, respectively).
  - To generate features with a particular correlation structure, we first divided the total number of features into bins of a prespecified size. In each bin, we generated one proxy feature to represent that bin. All the other features in that bin were generated in such a way that they are correlated to a certain degree with the representative feature. The representative feature was drawn from the standard Gaussian distribution. All the other features with the desired number of observations were also drawn randomly from the standard Gaussian distribution while being correlated with the representative feature. The degree of correlation between the representative feature and every other feature is based on a correlation coefficient that is randomly drawn from a pre-specified range of correlation coefficients.
  - We used the estimated beta distribution parameters (from step 1) to transform the Gaussian features into beta-distributed features for each of the standard Gaussian features. Briefly, the cumulative distribution function (CDF) of the Gaussian distribution is applied to each Gaussian feature to transform it into uniform random variables within the interval [0, 1]. Then, the percent-point function (PPF; inverse of CDF) of the beta distribution with specified beta distribution parameters is applied to each uniform random variable to transform it into beta random variables.
2. To have a setting of high-dimensional datasets without correlated features while still being sufficiently similar to the distributional properties of methylation data for apt comparison, we simulated methylation-like data without any dependencies between the features. For this, we followed the procedure described below:
  - We randomly sampled the desired number of methylation sites from a real-world experimental dataset (GEO accession number: GSE161651 [51, 52]) and estimated their beta distribution parameters (alpha and beta, respectively).
  - We use the corresponding estimated beta distribution parameters (from Step 1) for each simulated feature to draw the desired number of observations from the beta distribution.
3. To throw light on the type II error rates of all the controlling procedures simultaneously with the type I error rate, we simulated 1–2% of the total hypotheses being tested as false and are guaranteed to result in p-values < 0.05. For this, we used a subset of datasets from the large collection of datasets that we used to compare the behaviour of the Bonferroni and BH procedures, as shown in Fig. 1G in the main manuscript. We introduced effect sizes for a subset of features (between 1 and 2%) that align with a realistic number of significant hits reported in typical experimental studies. The effect sizes were introduced on the so-called *M*-values of methylation data (not in the beta value scale). All the features that were selected to have group differences were selected to have their mean *M*-values within a specified range before the introduction of effect sizes. Particularly, we chose all the features to have their mean *M*-values between 2 and 2.5. We added a constant value of 1 to one of the groups and verified that between 1 and 2% of the features indeed had *p*-values < 0.05 before adjustment for multiple comparisons.

#### Real-world datasets with shuffled labels

To investigate the FDR control on real-world high-dimensional datasets with a large degree of dependencies, we generated many semi-real-world methylation datasets, RNAseq datasets, metabolite datasets, and eQTL datasets using the plasmoid-like simulation strategy mentioned above [46–50]. A criterion for our investigation is that all the null hypotheses for statistical testing are true. For methylation, RNAseq, and metabolite data, we chose to shuffle the labels of real-world experimental datasets and divide the observations into two random groups with no known true differences. For eQTL data, we chose to keep the SNP data fixed while permuting the expression levels of observations. For methylation data, we used the dataset with GEO accession number GSE161651 [51, 52]. For RNAseq data, we used the dataset with GEO accession number GSE267625 [53, 54]. For metabolite data, we used urinary metabolite data measured using ^1^H-NMR from [55]. For eQTL data, we used publicly available data from [56] with GEO accession number GSE53261 [57]. One possibility is to use many real-world datasets from public databases as desired for our investigation (*n* = 10,000). However, without being uniformly processed and normalized in some form, potential experiment-specific and processing-specific biases of the different datasets can affect the conclusions of our investigation. To avoid this, we chose to repeatedly shuffle the labels of one real-world dataset. In methylation datasets, we sampled with replacement the number of desired features (often 10,000) to be comparable with our simulated methylation data. For RNAseq data, we used all the ~ 40,000 features. For metabolite data, we used all the ~ 65 features. For eQTL data, we did not exclude any observations or features from SNP or expression data.

#### Simulated multivariate normal features with and without dependencies

To avoid the intricate details of a two-group statistical test confounding our investigation on FDR control in high-dimensional datasets with dependencies, we chose to include an additional combination of high-dimensional datasets with different distributional properties and one-sample statistical testing. For this, we simulated datasets with multivariate normal features with dependencies following the identical procedure described in 1. b. For the comparative datasets without dependencies, we have drawn the desired number of observations from the standard normal distribution for each feature.

#### Statistical testing with quantification of FDR and FWER

For the two-group comparison statistical testing, we divided the observations randomly into two groups of equal size. We performed three different statistical tests: (a) a standard two-group Student’s *t*-test that assumes equal variances, (b) a moderated *t*-test (limma [58]) that is a slight variation of the standard *t*-test, where empirical Bayes methods are used to shrink the feature-wise standard deviation to the global standard deviation, and (c) a non-parametric Wilcoxon rank-sum test. For the one-sample statistical testing, we performed the Kolmogorov–Smirnov (KS) test to assess for normality of features. For real-world RNAseq data, we perform a standard differential expression analysis using DESeq2’s Wald test for negative binomial distribution data. For real-world metabolite data, log2-transformed and scaled metabolite data are analysed for group (randomly-assigned) differences using a standard *t*-test. For real-world eQTL data, we performed standard eQTL association analysis using MatrixEQTL R package [24] using linear models with population stratification as covariate and querying cis associations within 1 megabase proximity for every gene. We used the BH method [9] for controlling the FDR and the Bonferroni method [11, 12, 59] for controlling the FWER. When extending the comparison with various other FDR and FWER controlling methods, we used the following approaches: Sidak [26], Holm [27], Holm-Sidak [26, 27], Simes-Hochberg (SH) [28, 29], Benjamini-Yekutieli (BY) [30], two-stage approaches based on the work of Benjamini-Hochberg (TS_BH) [31], Benjamini, Krieger and Yekutieli (TS_BY) [32], and a resampling-based FDR controlling procedure similar to that of Yekutieli and Benjamini (resampling) [33]. All the compared methods were implemented in the multipletests module of statsmodels in Python, except for the resampling approach, which is not readily implemented either in Python or R to our knowledge. Thus, we implemented it ourselves. We used three different pre-specified nominal levels that are routinely employed in life sciences: 1%, 5% and 10%.

#### Simulation of datasets with varying dataset properties

For both types of high-dimensional datasets (beta and normally distributed), we generated many variations of the datasets with a variety of properties related to the sample size, number of features, and whether the features have dependencies or not. For datasets with dependencies, we also varied the total proportion of correlated features. For each variation of the parameters, we generated 10,000 datasets with identical properties. In addition, we also investigated the FDR control on 20,000 variations of real-world experimental datasets with dependencies (DNA methylation and RNAseq). In total, we investigated FDR control on 620,000 unique datasets. On each dataset, we performed different statistical tests (both parametric and non-parametric) and adjusted for both FDR and FWER at different pre-specified nominal thresholds, resulting in approximately 8 million unique FDR/FWER corrections.

#### Literature search to investigate the prevalence of various multiple testing correction methods in the presence of dependent test statistics

To investigate the prevalence of multiple testing correction methods in the presence of dependent test statistics, we performed a literature search using web scraping. We particularly focused on DNA methylation studies published from 2020 through March 2025 and analyzed the prevalence of three multiple testing adjustment procedures: BH, Bonferroni, and Benjamini-Yekutieli. The search process is detailed below.

We restricted our literature search to eight different journals that are popular avenues for publishing high-dimensional omics-based research, particularly studies based on epigenetics. Specifically, we searched Clinical Epigenetics, BMC Genomics, Genome Biology, Genome Research, NAR, Nature Communications, Nature, and Nature Genetics journals. To find all the relevant articles that are primarily investigating epigenetics, we first filtered articles that contain the epigenetic-related keywords mentioned below, either in the title or abstract. We then filtered for the presence of both the statistical testing-related keywords and multiple testing-related keywords either in the abstract or the full text. We then searched for the presence of specific multiple-testing adjustment procedure names or abbreviations. Reference sections were excluded to prevent false keyword matches based on the initials of the author names. Supplementary materials were not included in the web scraping process and were therefore not considered in the keyword-based filtering. We ensured that our keyword searches were case-insensitive, allowing us to detect relevant terms regardless of capitalization. Additionally, to account for plural forms, we applied pattern-matching techniques that recognized both singular and plural variations of keywords.

Epigenetic-related keywords included the following: “methylation,” “epigenetic,” or “epigenomic.” To determine whether articles employed statistical analyses, we searched for the following keywords: “statistical test,” “t-test,” “t test,” “anova,” “wilcoxon,” “kruskal–wallis,” “kruskal wallis,” “mann–whitney u,” “mann whitney u,” “mann–whitney–wilcoxon,” “mann whitney wilcoxon,” “wilcoxon mann whitney,” “wilcoxon-mann–whitney,” “rank-sum,” “rank sum,” “linear model,” “linear regression,” “limma,” “edgeR,” “deseq2,” “hypothesis test,” “*p*-value,” “*p* value,” “f-test,” or “f test.” To identify the articles that mentioned multiple testing adjustment-related keywords, we searched for “multiple testing,” “multiple comparison,” “multiple correction,” “multiple adjustment,” “multiple hypothesis,” “adjusted *p*-value,” “adjusted *p* value,” “family-wise error rate,” “family wise error rate,” “fwer,” “false discovery rate,” “fdr,” “q value,” or “q-value.”

After identifying articles that mentioned methylation, statistical analyses, and multiple testing corrections, we further checked for the presence of specific multiple testing correction methods: BH, Bonferroni, and Benjamini-Yekutieli (BY). To detect BH, we searched for the terms “benjamini-hochberg,” “benjamini hochberg,” and “bh.” Similarly, Bonferroni corrections were identified by the presence of the term “bonferroni.” Benjamini-Yekutieli was detected using “benjamini-yekutieli,” “benjamini yekutieli,” and additionally, a case-sensitive search for “BY.” The results were then recorded, allowing us to estimate how often specific correction methods were used and reported in methylation studies across the selected journals. To avoid cases where several correction methods were mentioned—potentially indicating benchmarking or comparative analyses—we only considered articles that referenced a single correction method.

## Supplementary Information

Additional file 1: Supplementary Figures S1-S3, Tables S1-S2 [65].

## Data Availability

The non-sensitive simulated data from this study (~610,000 unique datasets; approximately 9 terabytes) is permanently available at the following DOI: [10.11582/2024.00171](10.11582/2024.00171) [60] under an open-source Creative Commons Attribution 4.0 license. The intermediate datasets and analysis results of real-world experimental RNAseq and eQTL data are made permanently available under an open-source Creative Commons Attribution 4.0 license at the following DOI: [10.11582/2025.oa9vhljn](10.11582/2025.oa9vhljn) [61]. To ensure the ease of reproducibility, we containerised the whole computational environment necessary to replicate this study's findings. The Docker image of the computational workflow of this article is available from a public repository (Docker Hub) at [62]. The README file in the supplied GitHub repository describes a demo of the provided Docker image. All the source code and instructions to reproduce the simulations and findings of the study are available at GitHub under an open-source MIT license: [https://github.com/uio-bmi/fdr_under_dependencies](https://github.com/uio-bmi/fdr_under_dependencies) [63] and deposited in Zenodo with a permanent DOI [64]. Public experimental datasets used in this study are available elsewhere: methylation data [51, 52], RNAseq data [53, 54], metabolite data [55], and eQTL data [56, 57].
