# Supplementary material for: Beware of counter-intuitive levels of false discoveries in datasets with strong intra-correlations
Source: Genome Biol. 2025 Aug 18;26:249. doi: 10.1186/s13059-025-03734-z (PMC12359981; doi:10.1186/s13059-025-03734-z)
Supplement: Supplementary file 1 — Additional file 1: Supplementary Figures S1-S3, Tables S1-S2 [65]. [file 13059_2025_3734_MOESM1_ESM.docx]

Appendix


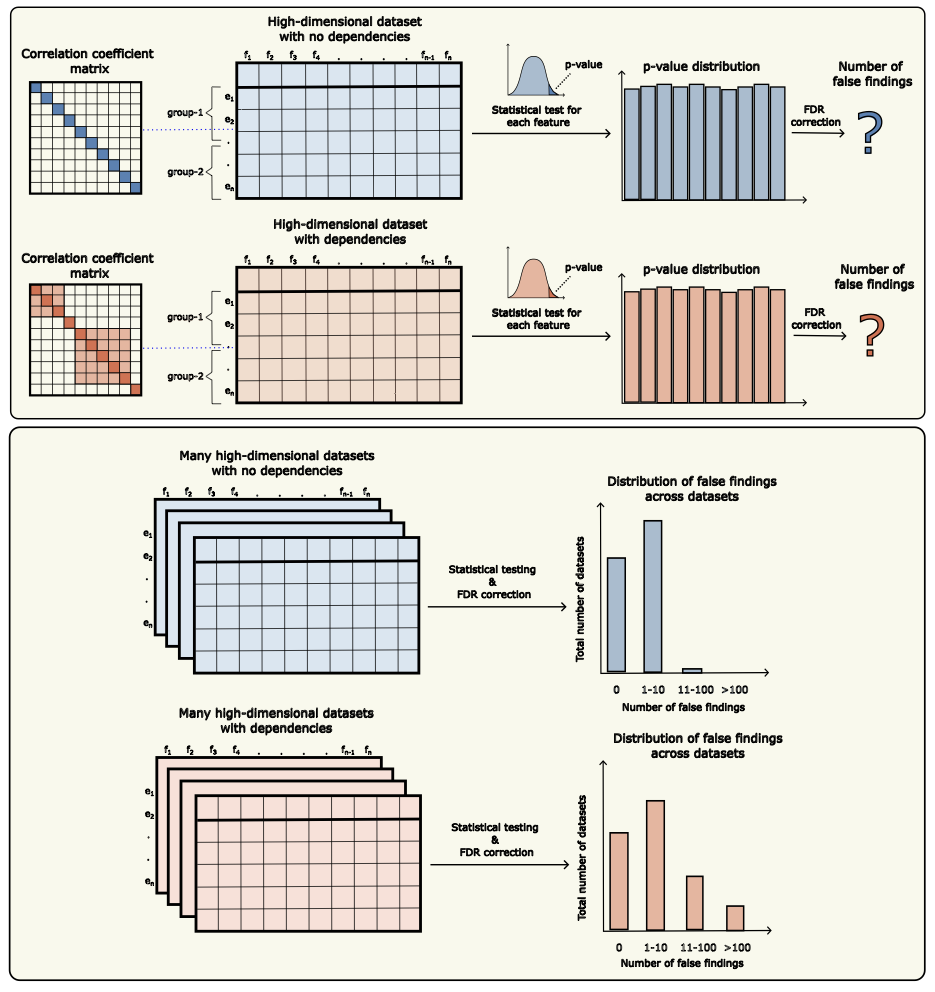


**Fig. S1** Graphical summary of the study design illustrating the influence of dependencies on the outcomes of multiple statistical hypothesis testing. **Top panel:** The workflow depicts the statistical hypotheses testing performed for each feature in the dataset (assuming all null hypotheses are true), followed by FDR correction. The false positive rate after FDR correction might vary depending on whether dependencies exist between features and to what extent. **Bottom panel:** Summary of the study findings demonstrating varying distributions of false findings after performing statistical testing followed by FDR correction across many datasets with and without dependencies. In scenarios with a high degree of correlations among features, we observed an increased frequency of an exaggerated number of false findings, underlining the effect of dependencies on multiple hypothesis testing outcomes.


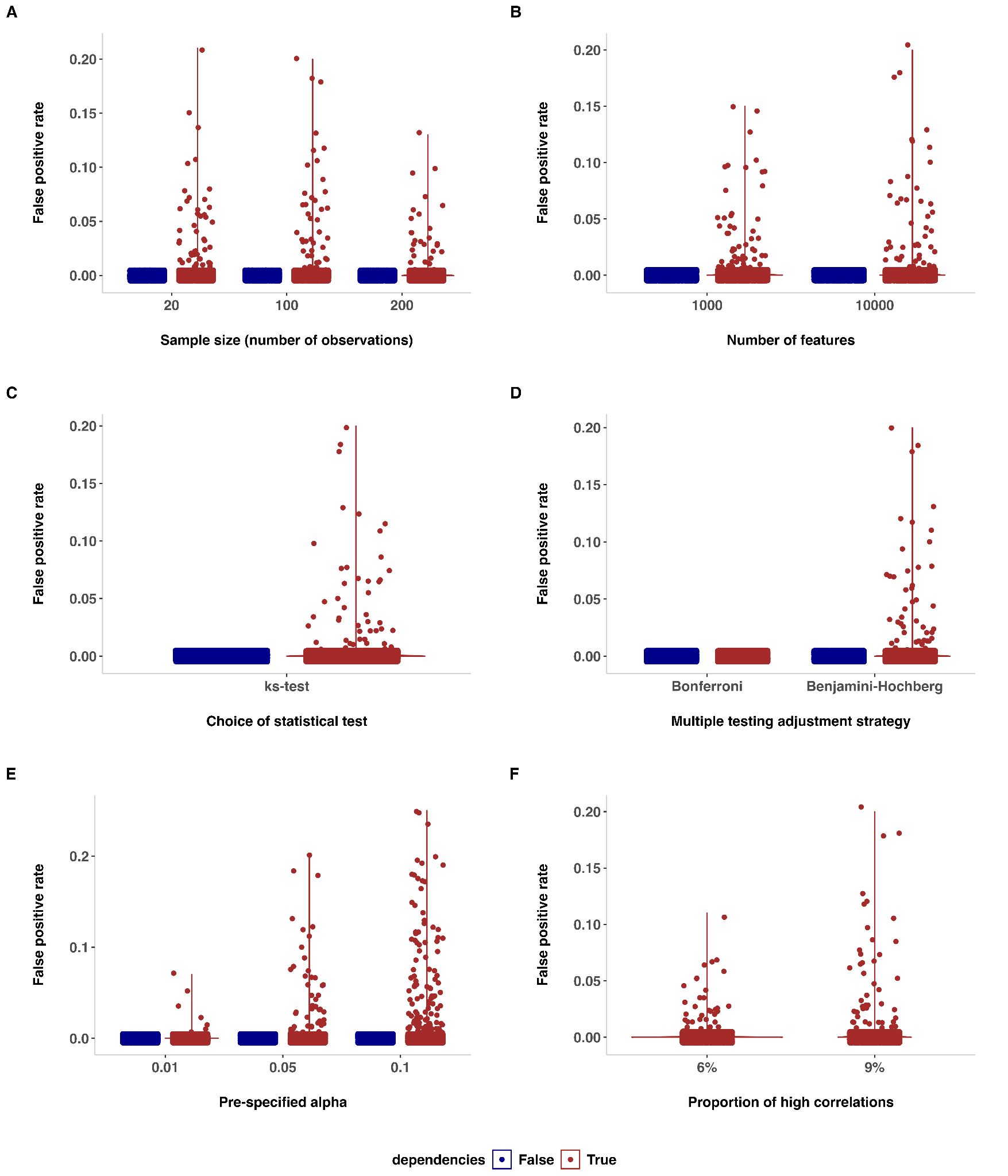


**Fig. S2 A-F,** Boxplots illustrating the impact of specific study design characteristics or analytical choices on the false positive rate across all 10,000 analysed datasets with and without dependencies (shown in different colors). We demonstrate the impact of the sample size (**A**), number of features (**B**), type of statistical test (**C**), FDR/FWER correction method (**D**), level of significance (alpha) (**E**), and degree of correlations between features (**F**). Note that here, the statistical hypotheses testing that was performed was a one-sample goodness of fit KS test followed by Benjamini-Hochberg correction at 5% FDR level. The number of observations was fixed at 100, and the number of features remained constant at 10,000 except when varying them for investigation.

# Supplementary Note 1

Many commonly encountered counting processes are well approximated by the binomial distribution, which assumes independence of the counted events. A property of this distribution is that the standard deviation is typically much smaller than the mean (close to the square root for rare events). This can lead to the development of an intuition that while the exact count for a process would vary if it were repeated, the relative variation is small, and the observed frequency is close to the true underlying probability of success. In the context of multiple statistical testing, this could translate to an intuition that the observed number of rejected tests would usually lie close to an underlying expected number of test rejections. However, with highly correlated data series, as is the case for DNA and methylation datasets, the variance becomes dramatically larger than that of the binomial distributions of the independence setting (**Figure S3**).

Using one representative dataset with dependencies (as in **Figure 1** from the main text), the p-values for the difference between groups naturally also become correlated between subsequent methylation sites (Pearson correlation coefficient (PCC in short) of 0.511). When considering whether the p-value is lower than a given threshold (here alpha=0.05) as a binary series, the correlation between subsequent positions is still strong (PCC of 0.342). Since the correlation of methylation values is quite local, the pairwise correlation between randomly located p-values drawn within the same dataset is only very slight (-0.009 for raw p-values and -0.002 for binarised values). Using a subset of 1000 simulated datasets with correlated methylation values (as in Figure 1 from the main text), we show that this dependency structure, however, increases the variance for the number of below-threshold p-values per dataset substantially (**Figure S3**). When controlling the family-wise error rate by applying the Bonferroni correction, the increased variance in the number of below-threshold p-values does not have any dramatic effect (**Figure S3**). The family-wise error rate is controlled (**Figure 1.G**, showing the number of rejected tests per dataset after Bonferroni correction). However, while also Benjamini-Hochberg (BH) succeeds in controlling the family-wise error rate (zero rejections for 96.4 % of the datasets), in the cases where a non-zero number of tests are rejected, the increased variance for the number of below-threshold p-values leads to a large variance in the number of rejections (see **Figure 1.A-C** and **Figure S3**, where the number of rejections after BH has a relatively wider distribution as compared to what an intuition inspired by binomial distributions would suggest).


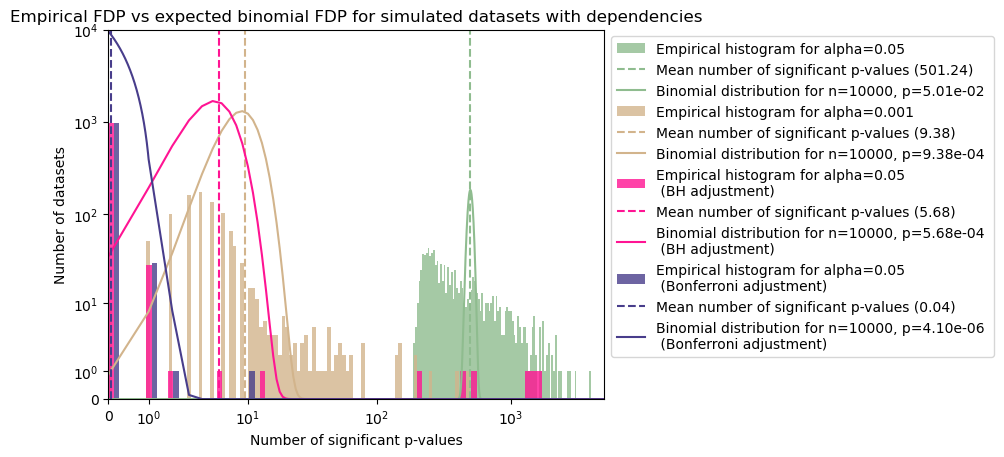


**Fig. S3** Histograms for the number of rejected features at different alpha levels and correction procedures based on 1000 simulated epigenetic datasets with feature dependencies. They are contrasted to binomial distributions at corresponding mean values that reflect the setting with independent tests. The variance for the number of rejections in the dependency setting is higher at every alpha level and further exaggerated by the BH correction (having a mean value of 5.7, but with individual histogram values beyond 1000). This high variance in the number of rejections by BH in the setting with all true nulls (where the BH procedure controls the false discovery rate [9]) bears similarities to the high variance observed for false discovery proportion across datasets at settings which include non-null features among the correlated features [65].

**Table S1:** Percentage of highly correlated features in the synthetic and real-world datasets. Synthetic datasets were simulated in two different variations to contain proportions of highly correlated features either (a) similar to or (b) somewhat higher than real-world datasets, where high correlation is defined as Pearson correlation coefficient > 0.4.

| **Dataset type** | **Percentage of highly correlated features**  **(Pearson’s Correlation coefficient > 0.4)** |
| --- | --- |
| real-world | 6.24 ± 0.06 |
| Synthetic (a) | 5.82 ± 0.1 |
| Synthetic (b) | 8.79 ± 0.18 |

##

##

##

**Table S2:** Statistics on the prevalence of BH, Bonferroni, and BY methods in eight different journals publishing omics-based investigations.

|  | Total epigenetic-relevant articles | Mentions a statistical test and multiple testing correction | Mentions none of the multiple testing correction methods searched for | Mentions at least one of the multiple testing correction methods searched for ^$^ | Mentions only Benjamini-Hochberg | Mentionsonly  Bonferroni | Mentions only  Benjamini-Yekutieli | Mentions >1 method |
| --- | --- | --- | --- | --- | --- | --- | --- | --- |
| BMC Genomics | 101 | 52 | 29 | 23 | 10  (43.48%) | 9  (39.13%) | 1  (4.35%) | 3  (13.04%) |
| Clinical Epigenetics | 660 | 295 | 171 | 124 | 16  (12.90%) | 95  (76.61%) | 4  (3.23%) | 9  (7.26%) |
| Genome Biology | 107 | 52 | 26 | 26 | 10  (38.46%) | 10  (38.46%) | 0  (0.0%) | 6  (23.08%) |
| Genome Research | 130 | 46 | 35 | 11 | 4  (36.36%) | 7  (63.64%) | 0  (0.0%) | 0  (0.0%) |
| NAR | 569 | 29 | 24 | 5 | 3  (60.0%) | 1  (20.0%) | 1  (20.0%) | 0  (0.0%) |
| Nature Communications | 827 | 546 | 314 | 232 | 97  (41.81%) | 105  (45.26%) | 2  (0.86%) | 28  (12.07%) |
| Nature | 173 | 113 | 87 | 26 | 5  (19.23%) | 20  (76.92%) | 0  (0.0%) | 1  (3.85%) |
| Nature Genetics | 132 | 73 | 41 | 32 | 2  (6.25%) | 26  (81.25%) | 0  (0.0%) | 4  (12.5%) |
| **Total** | 2699 | 1206 | 727 | 479 | 147 (30.69%) | 272  (56.99%) | 8  (1.67%) | 51 (10.65%) |

$ Used as the denominator for percentage calculations. The proportions are calculated relative to the filtered set of articles, which includes only methylation studies that applied a multiple correction method following a statistical test and mention at least one of the following: Benjamini-Hochberg, Bonferroni, or Benjamini-Yekutieli.
